# Retroviruses As Myeloid Cell Riders: What Natural Human Siglec-1 “Knockouts” Tell Us About Pathogenesis

**DOI:** 10.3389/fimmu.2017.01593

**Published:** 2017-11-21

**Authors:** Javier Martinez-Picado, Paul J. McLaren, Amalio Telenti, Nuria Izquierdo-Useros

**Affiliations:** ^1^IrsiCaixa AIDS Research Institute, Badalona, Spain; ^2^Institució Catalana de Recerca i Estudis Avançats (ICREA), Barcelona, Spain; ^3^University of Vic-Central University of Catalonia (UVic-UCC), Vic, Spain; ^4^National HIV and Retrovirology Laboratory, Public Health Agency of Canada, Winnipeg, MB, Canada; ^5^Department of Medical Microbiology and Infectious Diseases, University of Manitoba, Winnipeg, MB, Canada; ^6^Genomic Medicine, J. Craig Venter Institute, La Jolla, CA, United States

**Keywords:** antigen-presenting cell, human immunodeficiency virus type 1, Siglec-1, knockout, genome, human

## Abstract

Myeloid cells initiate immune responses and are crucial to control infections. In the case of retroviruses, however, myeloid cells also promote pathogenesis by enabling viral dissemination; a process extensively studied *in vitro* using human immunodeficiency virus type 1 (HIV-1). This viral hijacking mechanism does not rely on productive myeloid cell infection but requires HIV-1 capture *via* Siglec-1/CD169, a receptor expressed on myeloid cells that facilitates the infection of bystander target cells. Murine retroviruses are also recognized by Siglec-1, and this interaction is required for robust retroviral infection *in vivo*. Yet, the relative contribution of Siglec-1-mediated viral dissemination to HIV-1 disease progression remains unclear. The identification of human null individuals lacking working copies of a particular gene enables studying how this loss affects disease progression. Moreover, it can reveal novel antiviral targets whose blockade might be therapeutically effective and safe, since finding null individuals *in natura* uncovers dispensable functions. We previously described a loss-of-function variant in *SIGLEC-*1. Analysis of a large cohort of HIV-1-infected individuals identified homozygous and heterozygous subjects, whose cells were functionally null or partially defective for Siglec-1 activity in HIV-1 capture and transmission *ex vivo*. Nonetheless, analysis of the effect of Siglec-1 truncation on progression to AIDS was not conclusive due to the limited cohort size, the lack of complete clinical records, and the restriction to study only off-therapy periods. Here, we review how the study of loss-of-function variants might serve to illuminate the role of myeloid cells in viral pathogenesis *in vivo* and the challenges ahead.

Antigen-presenting cells (APCs) of the myeloid lineage trigger innate and adaptive immune responses against invading viruses, thus modulating the outcome, progression, and clearance of infections (1, 2). Yet, chronic viral infections counteract several defenses orchestrated by APCs and exploit immunity to favor persistence. Infection caused by the human immunodeficiency virus type I (HIV-1) is one of the best-studied examples to illustrate this paradox, where APCs act as a double-edged sword throughout the course of infection. Myeloid APCs (such as dendritic cells, monocytes, and macrophages) are not as susceptible to HIV-1 infection as activated CD4^+^ T cells (3). This is likely due to host restriction factors such as SAMHD1 (4, 5) that restrict viral infection and decreases myeloid cell capacity for immune sensing (6), limiting the onset of antiviral responses. However, HIV-1 can exploit myeloid APC biology to reach and infect new target cells through a mechanism that does not rely on the productive infection of myeloid cells. This process was described *in vitro* at the early nineties by the laboratory of Dr. Ralph Steinman (7), who received the Nobel Prize for discovering dendritic cells. Despite decades of research though, there is no convincing *in vivo* evidence that demonstrates whether myeloid APCs play a critical role in HIV-1 disease progression.

Upon cellular activation, myeloid APCs can capture and store large numbers of HIV-1 particles (8–10), which are then efficiently transferred to bystander CD4^+^ T cells (7, 11, 12) *via* cell-to-cell interactions established as part of their immune surveillance routine. Throughout this process of retention and release of virus, HIV-1 exploits a mechanism by which APCs acquire antigens transported by extracellular secreted microvesicles termed exosomes (13). The acquisition of exosomes by activated myeloid APCs contributes to antigen presentation to CD4^+^ T cells (14). This step helps to amplify adaptive immunity without the need for myeloid APCs to be in direct contact with the pathogen (15, 16). Retention of exosomes within intracellular compartments might serve as an antigen depot to control and sustain adaptive immune responses. However, in the case of HIV-1, this internalization route retains infectious particles within protected dynamic compartments (17–19) from where viruses are efficiently transmitted across infectious synapses to susceptible lymphocytes (7, 11, 12).

This particular mode of HIV-1 transmission is known as *trans*-infection; a route that favors *de novo* infection of target cells under circumstances where the same dose of cell-free-viruses do not establish productive infection (11). *Trans*-infection is largely dependent on the expression of the sialic-acid binding I-type lectin receptor Siglec-1 (CD169 or Sialoadhesin) (20–22). Siglec-1 is an interferon inducible receptor constitutively expressed on myeloid cells (23), that is highly upregulated upon myeloid APC exposure to antiviral type I interferons (24, 25). Siglec-1 is a trans-membrane receptor with a long neck that protrudes beyond the glycocalyx of the cell and a terminal V-set domain with the ability to interact with sialylated ligands. While the affinity of Siglec-1 for sialic acid-containing molecules is low, avidity for clusters of sialylated molecules is high (23), allowing for the specific recognition of packaged gangliosides that expose sialyllactose moieties on the viral membrane (26, 27). Likewise, Siglec-1 captures exosomes *via* recognition of sialylated gangliosides packaged on the microvesicle membrane (21), which assemble and bud from cellular membranes. Murine studies have also confirmed the capacity of Siglec-1 expressed on lymphoid tissues to capture exosomes *in vivo* (28). Pioneering reports suggested that DC-SIGN, a C-type lectin expressed on immature DCs that patrol peripheral mucosae in search of invading pathogens, could capture HIV-1 early after viral invasion, travel to lymphoid tissues, and establish productive CD4^+^ T cell infection *via trans*-infection (11). While C-type lectins such as DC-SIGN recognize the viral envelope glycoprotein (11), capture of HIV-1 *via* Siglec-1 is independent of this interaction (10, 21). Siglec-1 viral uptake largely exceeds the capacity of C-type lectin receptors for HIV-1 capture (21), making this process much more infectious and underscoring novel scenarios within secondary lymphoid tissues in which Siglec-1 *trans*-infection could fuel viral dissemination.

The molecular pathways governing HIV-1 *trans*-infection *via* Siglec-1 on APCs have been described *in vitro* using both monocyte-derived APCs (20–22) and primary myeloid cells directly isolated from human tissues (25) (Figure 1). Another retrovirus, the murine leukemia virus (MLV), also contains sialylated gangliosides and is captured *via* Siglec-1 *in vitro* (29). MLV exploits Siglec-1-mediated *trans*-infection of permissive lymphocytes to establish infection within secondary lymphoid tissues in mice (30) (Figure 1). However, the *in vivo* contribution of Siglec-1 to HIV-1 disease progression remains largely unknown.

**Figure 1:**
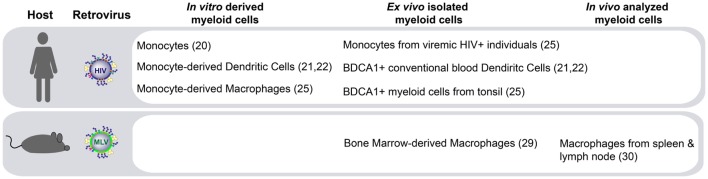
Siglec-1-mediated retroviral *trans*-infection on distinct myeloid antigen-presenting cells (APCs). Human immunodeficiency virus type 1 (HIV-1) capture *via* Siglec-1 and subsequent transfer to target cells has been reported not only in human APCs derived *in vitro* but also in activated primary myeloid cells isolated *ex vivo*. In murine models, Siglec-1 retroviral *trans*-infection has been reported *ex vivo*, and most importantly, due to the extraordinary ability of Siglec-1 positive APCs to capture cell-free viruses from the lymphatic vessels at the edges of the lymphoid tissue and their capacity to transfer that infectivity to permissive lymphocytes, this mechanism has also been observed *in vivo*.

The lack of available animal models to study HIV-1 infection makes it challenging to investigate the role of Siglec-1 on HIV-1 pathogenesis *in vivo*. Humanized mouse models susceptible to HIV-1 infection are only established in a few laboratories, and how disease progression in these animals correlates with human pathogenesis needs further investigation. Primate models are also restricted to specific facilities and rely on the use of primate retroviruses that may not directly reflect the biology of HIV-1. Under these circumstances, finding naturally occurring human knockouts could be a good alternative to address the role of key receptors such as Siglec-1 under physiological settings of infection. A deletion in the gene that codes for the HIV-1 co-receptor CCR5, which is needed for acquisition of CCR5-tropic HIV-1, is one of the best-known examples of how a genetic variant alters the phenotype of infection (31). These types of variants have been observed for decades and have also been confirmed by large-scale genomic analysis (32). However, these large-scale analyses have not uncovered novel candidates that might influence HIV-1 disease progression because the available sample sizes are not adequate to assess all possible classes of genetic variation, such as rare and low frequency polymorphisms.

The identification of individuals harboring rare, loss-of-function genetic variants provides an opportunity to study gene function *in vivo*. Recently, large catalogs of sequenced human genomes have demonstrated that individuals carrying homozygous loss-of-function variants, or natural human knockouts, can provide insight into genetic causes of disease and holds tremendous potential for identifying drug targets (33). However, given their generally low frequency, such variants have gone largely undetected in large-scale genomic analyses. As an alternative strategy to identify genes involved in HIV-1 progression, we conducted a search for individuals lacking the expression of Siglec-1 receptor to study the natural course of HIV-1 infection in the absence of this particular receptor (34). We focused on two well-established cohorts of HIV-1 infected individuals that had been longitudinally followed for decades and had extensive clinical records. We identified two homozygous and almost a 100 heterozygous subjects for a particular stop codon variant in the *SIGLEC1* gene. This stop-gain allele is found at highest frequency in individuals of European and South Asian ancestry (1.3%) and is rare or absent in African and East Asian populations (0.5%). *Ex vivo* experiments confirmed that cells from these individuals were functionally null or partially defective for Siglec-1 expression and, consequently, lost their activity in HIV-1 capture and transmission *in vitro*. While the lack of Siglec-1 is likely to abrogate *trans*-infection, the classical HIV-1 infection routes, including cell-free virus infection or cell-to-cell HIV-1 transmission still operate in the absence of Siglec-1, explaining the observation of Siglec-1 null individuals that are HIV-1 infected. However, despite the lack of impact on susceptibility, HIV-1 dissemination and disease progression in infected individuals with null or diminished Siglec-1 expression could be delayed compared with wild type individuals. Nonetheless, we did not observe an effect of Siglec-1 truncation on progression to AIDS.

Several challenges explain the lack of conclusive results in the study of Siglec-1 genetic variants (Figure 2). Power simulations indicate that analysis of a rare variant such as the Siglec-1 allele would require more than 10,000 individuals to detect a relative risk of 5 at *P* < 0.05 under a recessive model—an effect that would be similar to the beneficial outcome of B*57:01 on HIV-1 control (32, 35–37). This sample size far exceeds even the largest genome-wide studies of HIV-1 progression that comprises ~6,000 patients (38), which does not genotype the Siglec-1 stop variant and cannot be used to impute the presence of this rare allele. Given that the proposed effect requires long-term follow-up off therapy, it is extremely unlikely that a sufficient sample size could be reached to assess the long-term consequences of the Siglec-1 stop variant on HIV-1 disease. Another limitation faced was the lack of seroconversion date for most of the individuals screened; a clinical record that is normally missing in most cohorts of HIV-1 infected individuals. Thus, disease progression was only followed from the date of diagnosis, which may differ between individuals, especially if they are protected by beneficial phenotypes. Moreover, additional clinical data were missing from key individuals, even though we had focused on cohorts with exhaustive follow-up. Indeed, one of the homozygous individuals found had no clinical records for nine years, and information only resumed after antiretroviral treatment initiation, when viral suppression abrogated any potential effect that the Siglec-1 variant might have had on disease progression. Since current clinical guidelines recommend treatment introduction early after HIV-1 diagnosis (39), in the near future this type of analyses will be restricted to retrospective cohorts, which followed old recommendations and started treatment when CD4 counts dropped below a certain threshold, offering a window of opportunity to monitor the natural course of infection. Finally, complexity also arises from the analysis of phenotypes that can be influenced by the infection of several pathogens at the same time. Indeed, one of the homozygous individuals for the rare Siglec-1 allele had a high CD4^+^ T cell count that dramatically dropped when tuberculosis was diagnosed. Lack of Siglec-1 could have had a negative impact on the immune control of the mycobacterial infection, masking any putative beneficial effects caused during HIV-1 progression. Previous studies indicate that Siglec-1 expression on myeloid APCs has a role in combating sialylated bacteria (40, 41). Although sialylation of *Mycobacteria* has not been documented to our knowledge, direct interaction between Siglec-1 and *Mycobacteria* might not be required to impact antibacterial immunity. Alternatively, the lack of Siglec-1 on myeloid APCs could compromise antigen capture *via* exosome or microvesicle transfer and affect the control of the bacterial infection (42–45).

**Figure 2:**
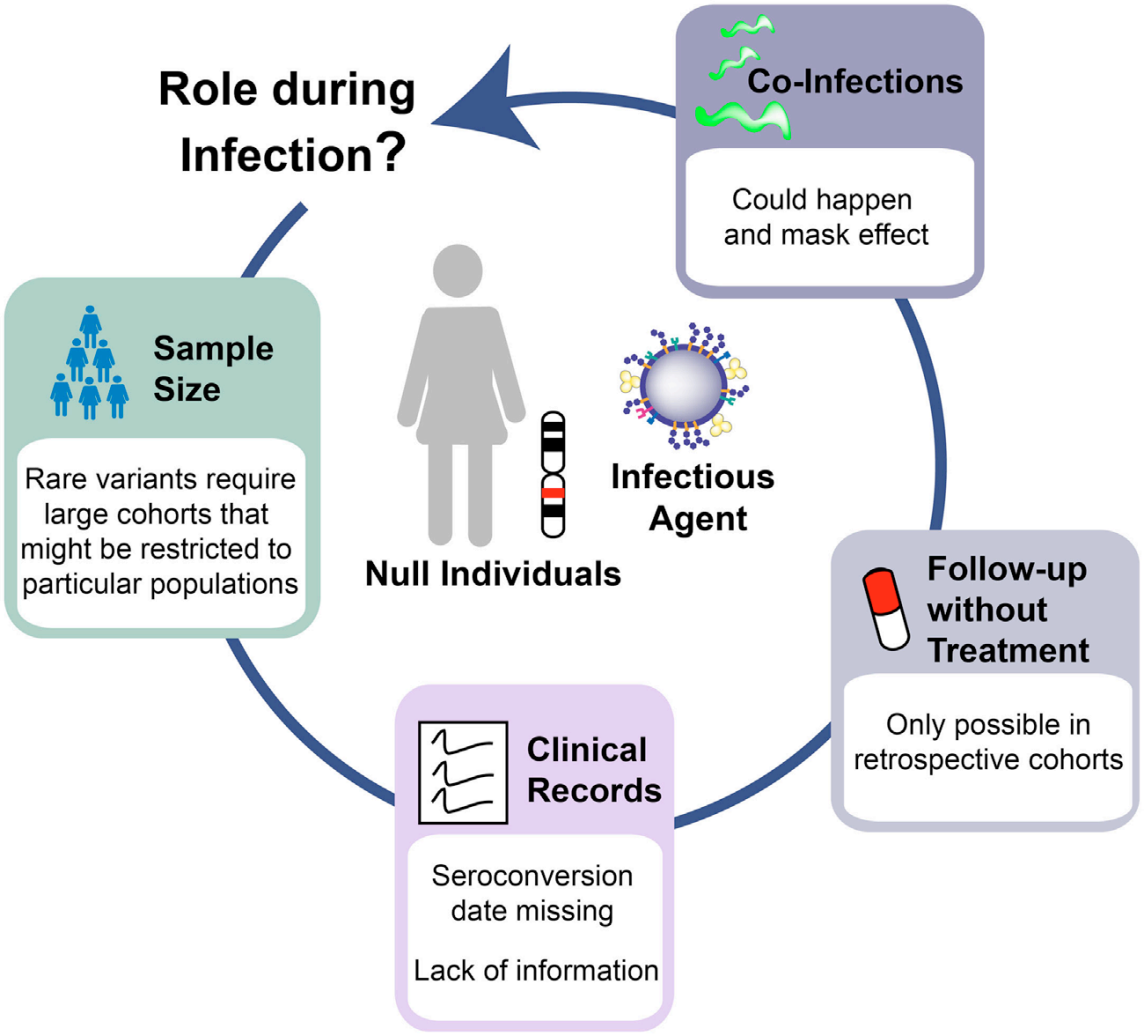
Challenges to interpret human knockout genetics in infectious diseases. The study of Siglec-1 null individuals infected with human immunodeficiency virus type 1 highlights the barriers to understand the *in vivo* role of human knockout genes. Limitations arises from: the need to study large cohorts that can be restricted to specific populations where the variant of interest has the highest frequency; the lack of critical clinical information even in cohorts with good follow-up; the introduction of therapies that unmask any putative effect of the studied variant; and the co-infection with additional pathogens that might influence the observed phenotype in the opposite direction from what is expected.

Overall, difficulties and questions faced throughout the study of Siglec-1 null individuals infected with HIV-1 illustrate the major challenges of the field of human knockout genetics applied to infectious diseases. We need to address the biological function of knockout genes of interest *in vivo* and the effect of a particular variant on health-related phenotypes (46). Variability in the observed phenotypes arises not only from the effect that other genetic variants might have on the gene of interest, but also from the exposure to particular environmental conditions, including the co-occurrence of infections. Animal studies could help to dissect the contribution of these factors by creating the same genetic background and similar environmental conditions in pathogen-free facilities, where co-infections could be experimentally controlled. Working with adequate animal models is, however, complex in the case of HIV-1. An interesting alternative to unambiguously test the potential contribution of myeloid APCs *via* Siglec-1 to HIV-1 disease progression could be to develop antiviral therapeutic agents against Siglec-1. The identification of Siglec-1 null individuals demonstrates that this protein is dispensable, and its therapeutic blockade is therefore expected not to cause serious side effects. Future work targeting Siglec-1 could provide conclusive evidence of the real contribution of myeloid APC to HIV-1 pathogenesis *in vivo*. If proven effective, this new family of antiviral agents against HIV-1 could also offer protection against other retroviral infections by mimicking the loss-of-function mutation found in *SIGLEC1*.

## Author Contributions

JM-P, PM, AT, and NI-U designed the work, prepared the figures, reviewed bibliography, and prepared the manuscript. All the authors approved the final version.

## Conflict of Interest Statement

The authors declare that the research was conducted in the absence of any commercial or financial relationships that could be construed as a potential conflict of interest.

## Abbreviations

APCs, antigen-presenting cells; HIV-1, human immunodeficiency virus type 1; MLV, murine leukemia virus.
